# Seeing the results of a mutation with a vertex weighted hierarchical graph

**DOI:** 10.1186/1753-6561-8-S2-S7

**Published:** 2014-08-28

**Authors:** Debra J Knisley, Jeff R Knisley

**Affiliations:** 1 Department of Mathematics and Statistics, East Tennessee State University, Johnson City, TN 37614; 2 Institute for Quantitative Biology, East Tennessee State University, Johnson City, TN 37614

## Abstract

**Background:**

We represent the protein structure of scTIM with a graph-theoretic model. We construct a hierarchical graph with three layers - a top level, a midlevel and a bottom level. The top level graph is a representation of the protein in which its vertices each represent a substructure of the protein. In turn, each substructure of the protein is represented by a graph whose vertices are amino acids. Finally, each amino acid is represented as a graph where the vertices are atoms. We use this representation to model the effects of a mutation on the protein.

**Methods:**

There are 19 vertices (substructures) in the top level graph and thus there are 19 distinct graphs at the midlevel. The vertices of each of the 19 graphs at the midlevel represent amino acids. Each amino acid is represented by a graph where the vertices are atoms in the residue structure. All edges are determined by proximity in the protein's 3D structure. The vertices in the bottom level are labelled by the corresponding molecular mass of the atom that it represents. We use graph-theoretic measures that incorporate vertex weights to assign graph based attributes to the amino acid graphs. The attributes of the corresponding amino acids are used as vertex weights for the substructure graphs at the midlevel. Graph-theoretic measures based on vertex weighted graphs are subsequently calculated for each of the midlevel graphs. Finally, the vertices of the top level graph are weighted with attributes of the corresponding substructure graph in the midlevel.

**Results:**

We can visualize which mutations are more influential than others by using properties such as vertex size to correspond with an increase or decrease in a graph-theoretic measure. Global graph-theoretic measures such as the number of triangles or the number of spanning trees can change as the result. Hence this method provides a way to visualize these global changes resulting from a small, seemingly inconsequential local change.

**Conclusions:**

This modelling method provides a novel approach to the visualization of protein structures and the consequences of amino acid deletions, insertions or substitutions and provides a new way to gain insight on the consequences of diseases caused by genetic mutations.

## Background

Historically, graphs have been used to represent chemical structures since the inception of graph theory and there is a well-developed field known as chemical graph theory [1,2]. Whereas in chemical graph theory each vertex represents an atom, the size of a protein molecule does not lend itself well to this representation. Thus, in many cases in the literature where a protein is represented by a graph, each vertex represents an amino acid and therefore each vertex represents ten, more or less, atoms. Two vertices are connected by an edge in the graph if the corresponding amino acid residues are within a specified distance threshold, typically 7 or 8 angstroms. Using this approach, protein structures can be viewed as networks of amino acids [3-6]. Even so, due to the size of many proteins, these graphs still tend to be very large. Since many of the chemical descriptors are defined for small molecules, measures from network science proved to be more informative for macromolecules.

Topological features of protein structures exhibit many desirable network properties such as high clustering coefficients and short average path lengths which shed light on aspects of protein folding [7-9]. A review of the uses of graphs as models of protein structure can be found in [10], which is a summary of work prior to 2002 for each method of representation - that is, vertices representing atoms versus vertices representing amino acids. However, again due to the size of these graphs, graph-theoretic representations have not provided an effective visualization tool, nor has it been an effective way to determine other properties of the protein's structure, such as the location of binding sites. In order to address the challenge of modelling a molecule at different scales - that is, simultaneously capturing full scale global properties of a large graph while identifying local properties of a small region of the graph - we developed a vertex weighted hierarchical graph model of a protein structure [11]. There are distinct advantages to each of the approaches discussed above, namely to let each vertex represent an atom, or to let each vertex represent an amino acid. To capture the advantages of each, we build a representation that uses both methods. That is, we use the nested graphs concept to integrate the information obtained at each scale with all other scales. To do so we construct a hierarchical graph-theoretic structure to represent the three-dimensional structure of a protein. Since the goal in [11] was to identify the global effects of a single point mutation, we now address the BioVis Data Challenge with the vertex-weighted, hierarchical graph approach.

We first represent the challenge protein scTIM as a graph with 19 vertices. Each vertex represents a substructure loosely determined by the secondary structures of the protein. Each substructure contains either a beta strand or an alpha helix, but not both. For example, substructure D1 contains a single beta strand and substructure D2 contains a single alpha helix. Other secondary structures such as loops and turns are not strictly separated, although they may be if the loop is exceptionally long as in the case of D14. The 19 substructures are given in Table 1.

**Table 1:** Substructure intervals used for the midlevel graph.

Substructure name	sequence location	substructure sequence
D1 -beta	2-15	ARTFFVGGNFKLNG
D2 - alpha	15-30	GSKQSIKEIVERLNTA
D3 - beta	30-43	ASIPENVEVVICPP
D4 - alpha	43-55	PATYLDYSVSLVK
D5 - loop	55-74	KKPQVTVGAQNAYLKASGAF
D6 - alpha	74-88	FTGENSVDQIKDVGA
D7 - beta	88-96	AKWVILGHS
D8 - alpha	96-105	SERRSYFHED
D9 - alpha	105-120	DDKFIADKTKFALGQG
D10 - beta	12-130	GVGVILCIGET
D11 - alpha	130-139	TLEEKKAGKT
D12 - alpha	139-151	TLDVVERQLNAVL
D13 - beta	151-166	LEEVKDWTNVVVAYEP
D14 - loop	166-177	PVWAIGTGLAAT
D15 - alpha	177-204	TPEDAQDIHASIRKFLASKLGDKAASEL
D16 - beta	204-211	LRILYGGS
D17 - alpha	211-225	SANGSNAVTFKDKAD
D18 - beta	225-237	DVDGFLVGGASLK
D19 - alpha	237-248	KPEFVDIINSRN

**Figure 1:**
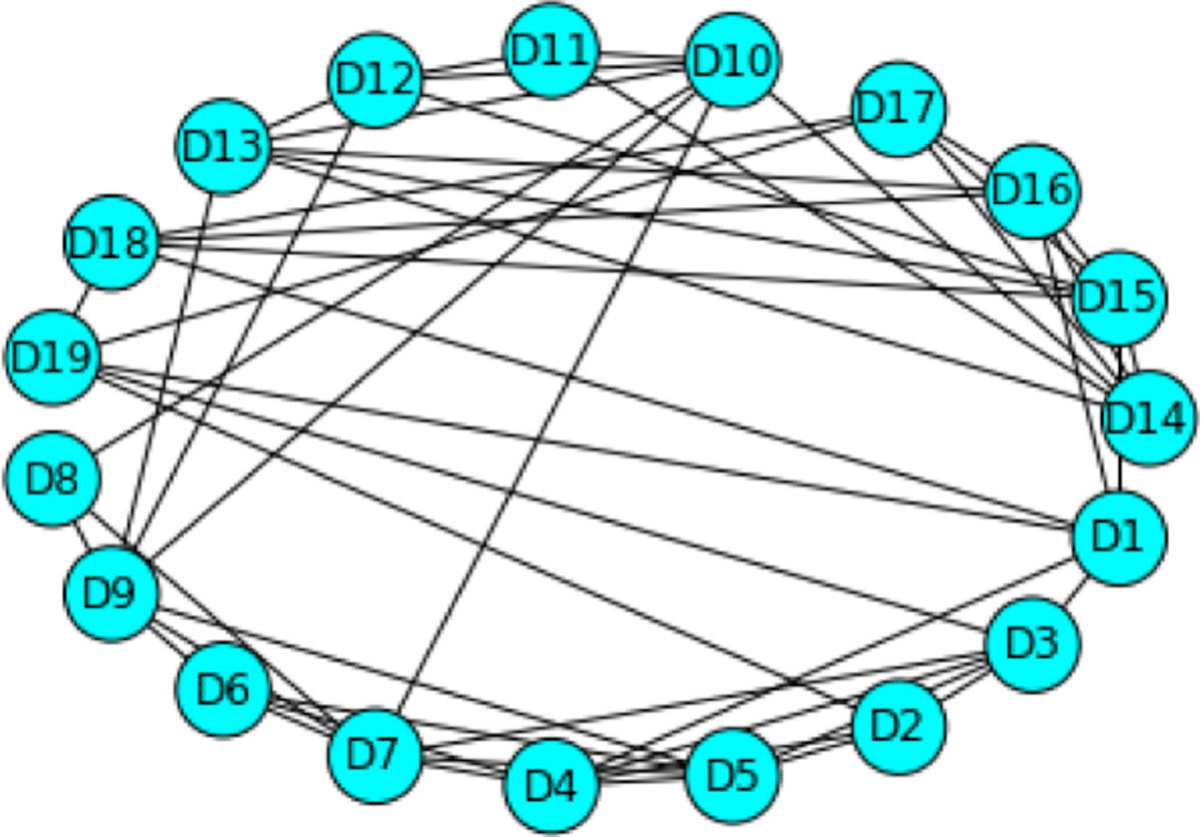
**The top level graph for scTIM**. Graphs D1 to D19 are the midlevel graphs representing each of the 19 substructures, respectively. Properties of the midlevel graphs are quantified by the vertex weights in the top level graph. Secondary structural elements are nodes, and two nodes are connected by an edge if there are a pair of amino acids in the domains that are separated by no more than 7.0 A.

We label the vertices Di, 0 < i < 20 and two vertices, Di and Dj are connected if there exist at least two pairs of amino acids whose distance does not exceed 7 angstroms where for each pair, one amino acid is in Di and one is in Dj. We call this graph the top level graph. The top level graph is shown in Figure 1. In turn, each substructure of the protein is also represented as a graph. That is, nested in each vertex of the top level graph is another graph. This collection of nested graphs constitutes the midlevel graphs. For graphs at the midlevel, each vertex represents an amino acid and two vertices are adjacent if the distance from the central carbons of each residue is not more than 7 angstroms. In addition, this must hold true for at least two amino acid pairs. We use the data in the Protein Data Bank [12] file 2YPI for these calculations. Other criteria can be applied. For instance, we have sometimes measured from the centroid of the residues and used a distance threshold of 8 angstroms.

Finally, each amino acid is represented by a graph where each vertex represents an atom and two vertices are adjacent if the corresponding atoms have a bond. We do not consider the hydrogen atoms in this model (this is the commonly known hydrogen suppressed ball-and-stick representation of a molecule). We let a single vertex represent the central carbon in the backbone and thus we obtain a rooted graph where we denote the backbone carbon as the root. There are twenty graphs at this level which we can refer to as the bottom level. More layers are possible and may be desirable for a very large protein or a protein complex. Thus this general method can be applied to a very large structure such as a protein complex or a relatively small protein. We determined that three layers are sufficient for the scTIM model.

We first describe the process for a single point mutation. Associated with each amino acid is a set of descriptors derived from graph-theoretic measures of its graph representation. These values were first defined by the authors in [13] where a neural network was trained to recognize a change in binding affinity due to mutations. A single point mutation in a protein results in a change in exactly one of the amino acids in the protein's amino acid sequence. Thus, one vertex in exactly one of the midlevel graphs will receive a new set of descriptors that corresponds to the change at the bottom level. This results in a change of attributes of a single vertex at the midlevel. This change of attributes at the midlevel results in a change of graph-theoretic measures of the midlevel graph that utilize vertex weights. Consequently, the vertex at the top level graph that represents the substructure where the single point mutation occurred will receive a new set of attributes. In this way we are able to capture the flow of information from a single point mutation to the entire protein and visualize the effects. The process described above changes the vertex weights, but not the structure itself, of the top level graph. Using graph-theoretic measures that incorporate vertex weights, we obtain a unique set of graph-theoretic values associated with each mutation.

In addition, there are a number of folding algorithms that will provide a predicted structure for a given amino acid sequence. For example, PhYre^2 ^[14] and I-TASSER [15] have both consistently performed well in the annual protein prediction competition known as CASP- Critical Assessment of Structure Prediction [16]. Consequently, a predicted pdb file can be obtained for a mutated sequence and the process described above can be iterated with the predicted structure. This results in a top-level graph whose structure may differ from the wild type. For the life scientists, we are developing a tool for "virtual mutations". By changing a residue (or a set of residues) in the sequence, this changes a vector of descriptors for a vertex (or vertices) at the midlevel. This in turn changes the values of the Top Level graph that are associated with that mutation. The consequences of the mutation on the structure of the graph can be viewed immediately. We describe the methods in more detail in the methods section and the results are below.

## Results

### Modelling a single point mutation with a predicted structural change

**Figure 2:**
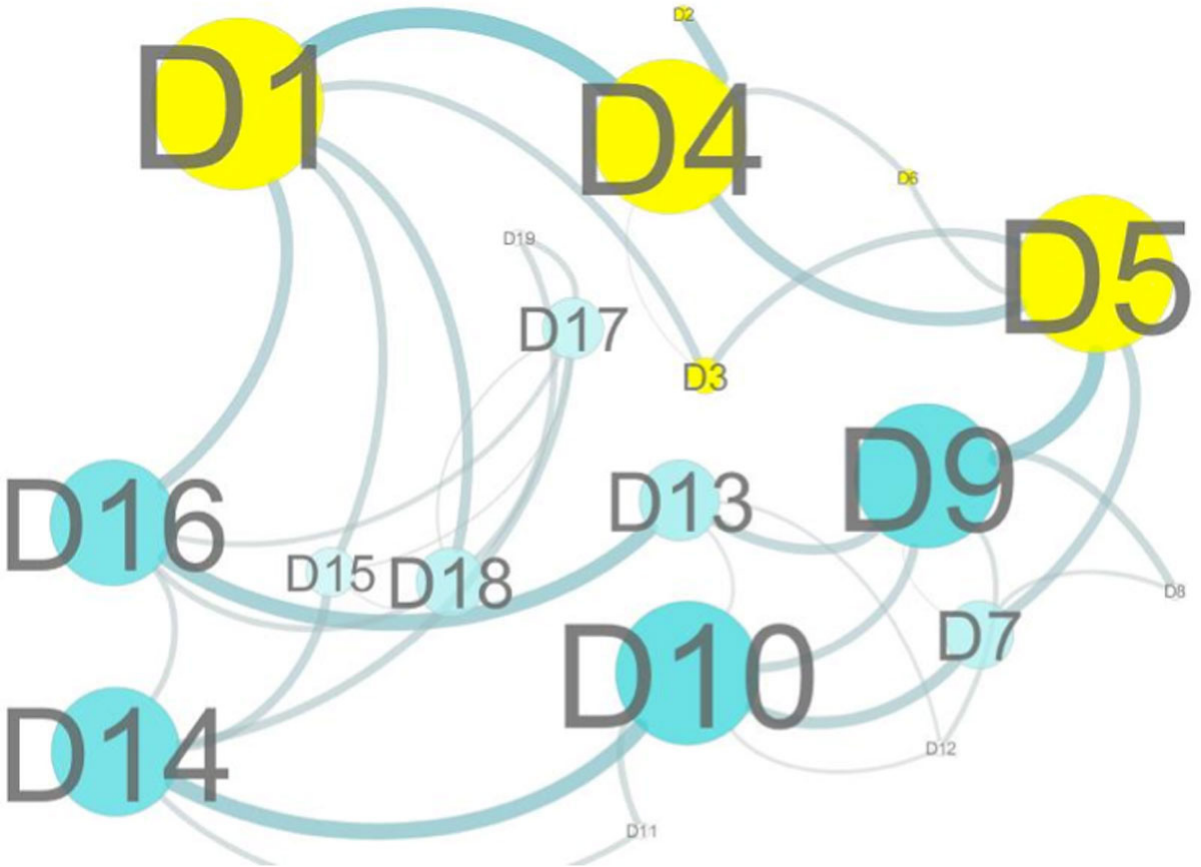
**Top level graph with no mutations**. Cytoscape visualization of the top level graph whose vertex weights were determined by properties of the midlevel graphs, which in turn were weighted by amino acid descriptors. By mapping additional bioinformatic and biophysical properties to the nodes and edges, and allowing these weights to guide the layout, we can acquire additional intuition into the importance in different domains and domain-domain interactions.

**Figure 3:**
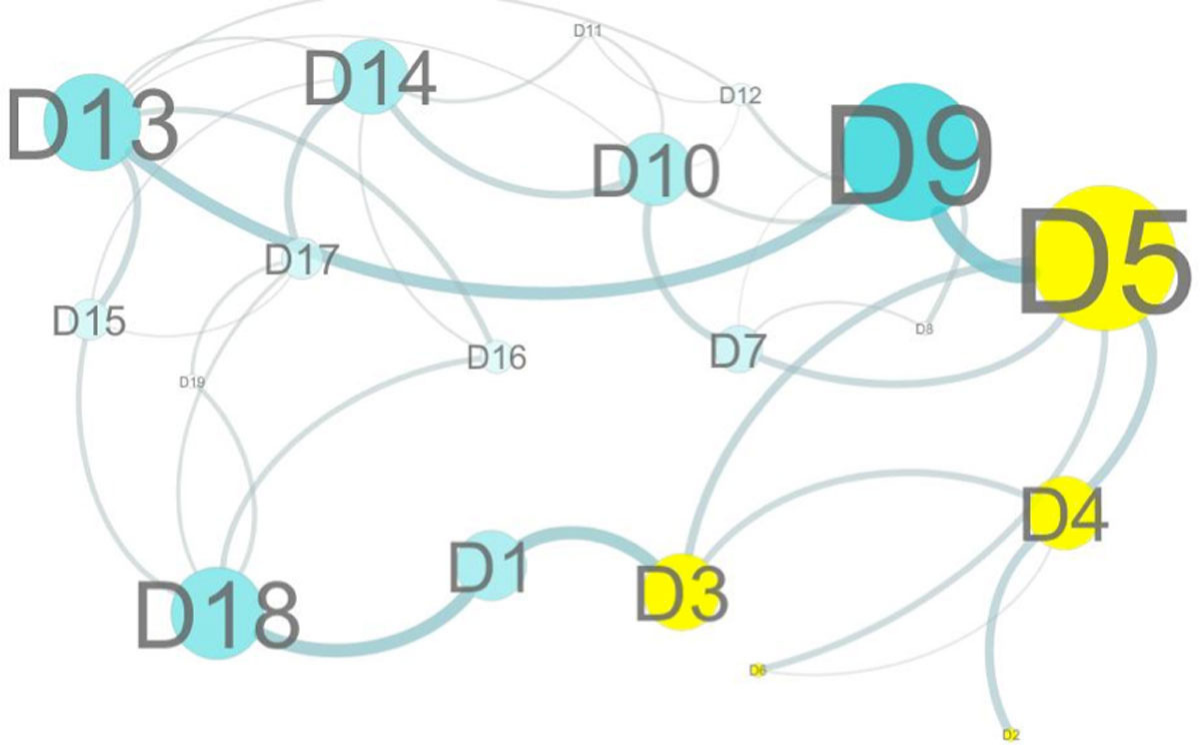
**Top level graph with V51R**. Cytoscape visualization parallel to figure 2, with the V51R mutation, which occurs in D4. Applying the same approach and weighting schemes to a different protein, here a hypothetical protein mutant, we can see how the mutation affects the interactions that are important in the wild-type protein.

First we show the result when the vertex weighted hierarchical method is coupled with a predicted change in the 3D structure. Figures 2 and 3 show the top level graph of the wild type protein and the mutant protein V51R respectively and were generated by Cytoscape [17]. We selected this mutation, V51R, to illustrate how a single point mutation can affect the top level graph in a significant way. The single point mutation V51R is one of the mutations found in the defective protein dTIM provided by the contest designers. To obtain these figures, we submitted the mutant sequence to PhYre^2^. PhYre^2 ^returns the predicted structure as a pdb type file. We construct the hierarchical graph for each, the predicted structure provided by PhYre^2 ^and the wild type provided by the PDB file 2YPI. To determine the vertex weights at each level, we can begin with the bottom level. Associated with each amino acid graph are a number of graph-theoretic measures such as the weighted domination number and the weighted degree of a vertex. To define the graph based measures, we modify the definition of common graphical invariants such as those found in a standard introductory text for graph theory [18,19]. For example, the *degree of a vertex v *in a graph is the number of neighbors of *v*. For a vertex-weighted graph, we define the *weighted-degree of a vertex v *as the sum of the weights of the neighbors of *v*. Note that in a standard graph without vertex weights, if we assign all vertices a weight of one, then the weighted definition is equivalent to the standard definition. Thus weighted definitions generalize the standard definitions in a natural way. For instance, we can also generalize the standard definition of the domination number of a graph.

A vertex set *S *is said to be a *dominating set of vertices *if every vertex in the graph is either in the set *S *or has a neighbor in *S*. Necessarily then, the entire set of vertices of a graph is a dominating set. The *domination number of a graph *is the minimum cardinality among all dominating sets. Since the cardinality of a set can be found by assigning a weight of 1 to each element of the set and then summing the weights, we define the weighted-domination number to be the minimum weight among all dominating sets. The *maximum degree of a graph *is the maximum value among all degree measures in the graph. Whereas the degree of a vertex is a local measure, the maximum degree of a graph is a global measure. Almost all standard graphical measures thus lend themselves to a "weighted" version. Graph-theoretic measures such as the maximum weighted-degree provide a rich source for numerical characterizations for the mid-level graphs which in turn are weights for the vertices of the top level graph. By including the vertex weights, we show the graph for the top level graph below generated by Cytoscape. Figure 2 is the wild type and Figure 3 is the mutant. Substructure D4, where the mutation occurred, is highlighted together with the neighbors of D4. Notice that there are significant structural changes predicted by PhYre^2^, such as the loss of the edge connecting D1 with D4. However, without the vertex weights, the change would not be nearly as striking.

Using this approach, the effects of a single point mutation on the entire protein can be observed. One would expect that the vertex representing the substructure where the mutation occurred to be affected. However, other consequences can now also be observed, such as the loss of the "heavy" cycle, D1, D4, D5, D9, D13, D16. The differences and similarities between a wild type protein and a given mutation of that protein are difficult to discern in a model where each vertex represents an amino acid. Without the hierarchical structure, a change in a single amino acid (a single vertex) may seem inconsequential, especially among the hundreds of such changes possible. With the hierarchical structure, however, the influence of such mutations can be tracked at each scale of representation.

### Modelling mutations without a change in predicted structure

**Figure 4:**
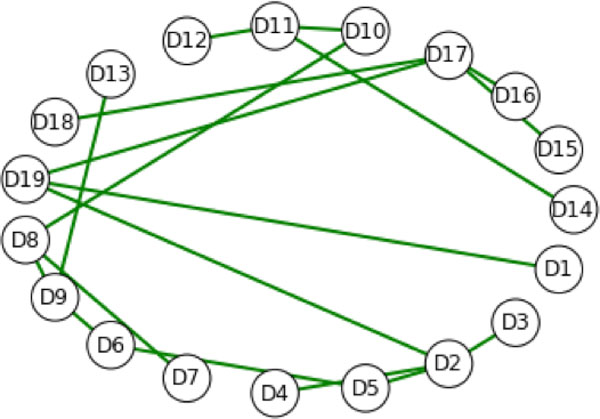
**The hydrophathy minimum spanning tree for the scTIM**. The edges of the top level graph (Figure 1) are weighted by the sum of the respective vertex weights. Each vertex is weighted by the maximum over the hydropathy weighted degrees of the corresponding midlevel graph. The complete top level graph can be filtered based on different properties of interest, here hydropathy, to identify likely physical causes for mutation effects.

Typically, the goal is to capture the top level impact of all the mutations in a given residue sequence. Figure 4 illustrates the process for producing such visualizations without utilizing a predicted change in the structure. In this method, the edge set of the top level graph does not change, only the vertex weights. One method for visualizing the effects in this case is to weight each edge {Di, Dj} in the top level graph with the average value of the vertex weights for Di and Dj. Subsequently, changes in descriptor weights can imply changes in the minimum spanning tree(s) of a top level graph. A *spanning tree of a graph G *is a graph with the same vertex set of G with the minimum number of edges that can be selected from the edge set of G so that G remains connected. For a given connected graph G, if G is a tree, then it has only one spanning tree, namely itself. Otherwise, if G is a connected graph that has more edges than a tree, it may have many spanning trees. When the edges are weighted, then the minimum spanning tree is the spanning tree whose edge sum is minimal. The famous Traveling Salesman Problem is an illustration of an application of the minimum spanning tree concept.

**Figure 5:**
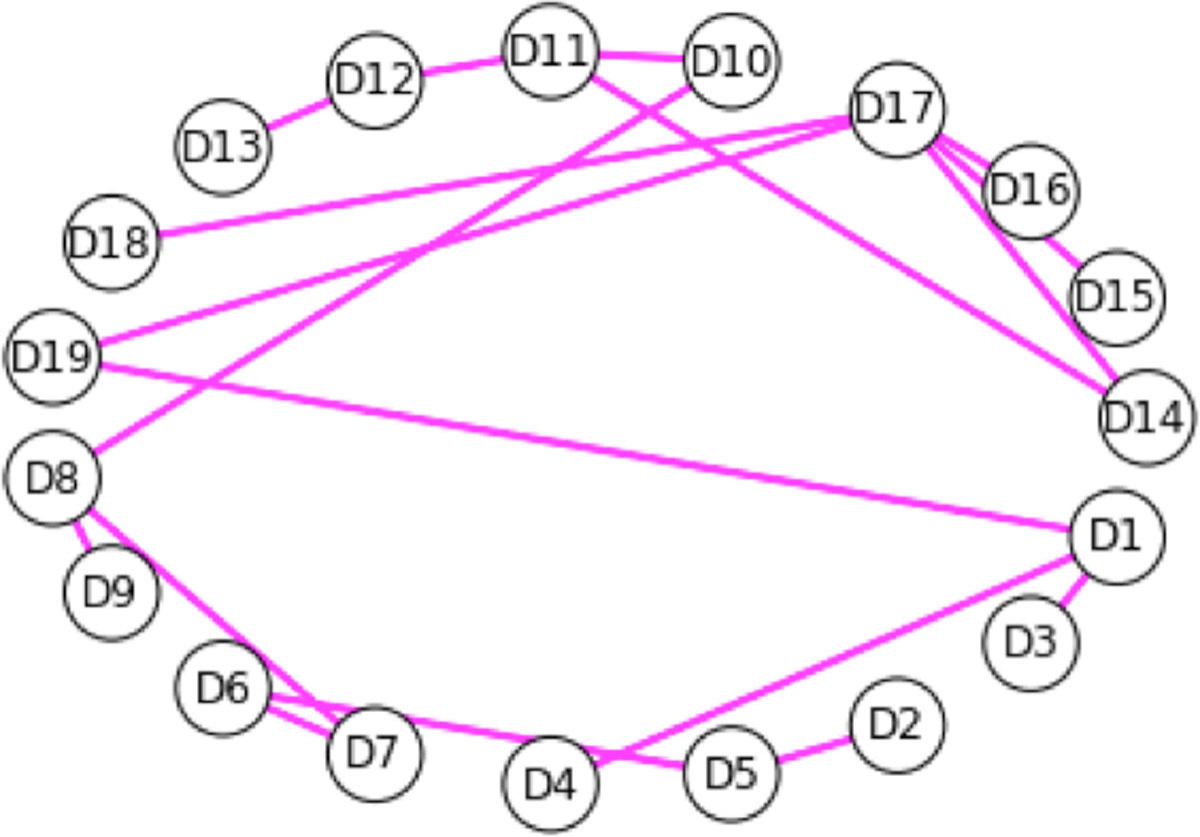
**The hydropathy minimum spanning tree for dTIM**. Using the sequence dTim, the weighting scheme and descriptors are the same as Figure 4. When the same minimum spanning tree is produced for the structurally similar scTIM, it is clear that the hydropathy-based interactions are significantly perturbed in the mutant vs. the wild-type protein.

**Figure 6:**
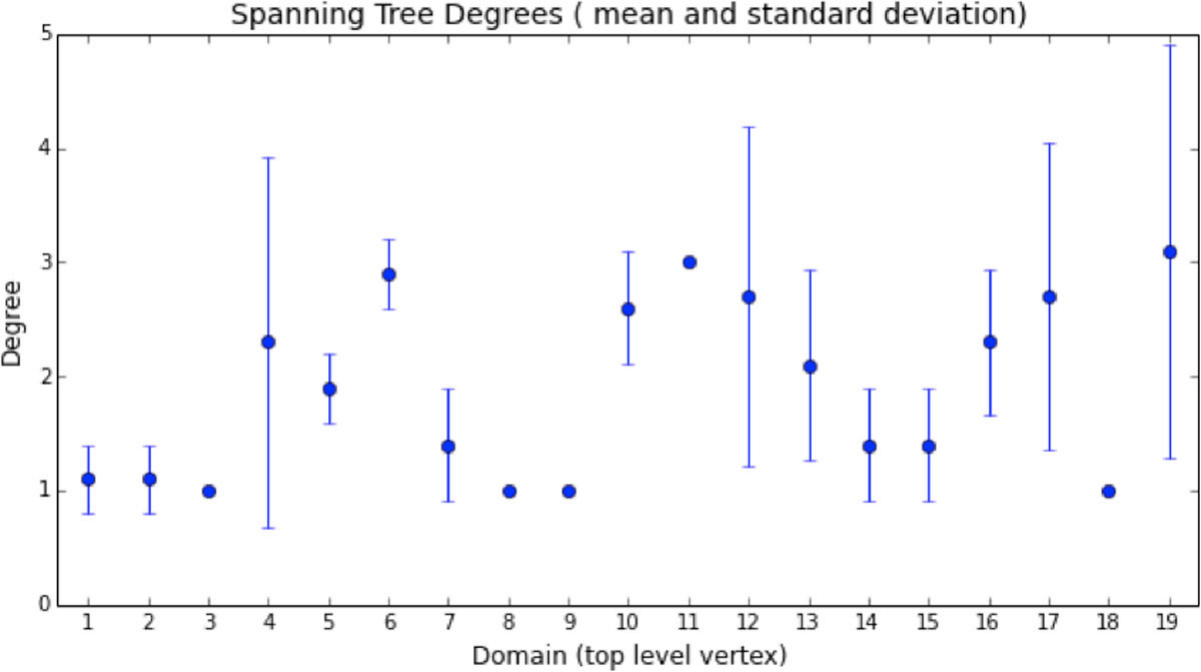
**Top level vertex mean degree (point) with standard deviation (error bar)**. Mean spanning tree vertex degrees across all mutations and wild type. The top level vertices (midlevel substructure graphs) corresponding to D3, D8, D9, D11, and D18 are not changed by any of these mutations.

**Figure 7:**
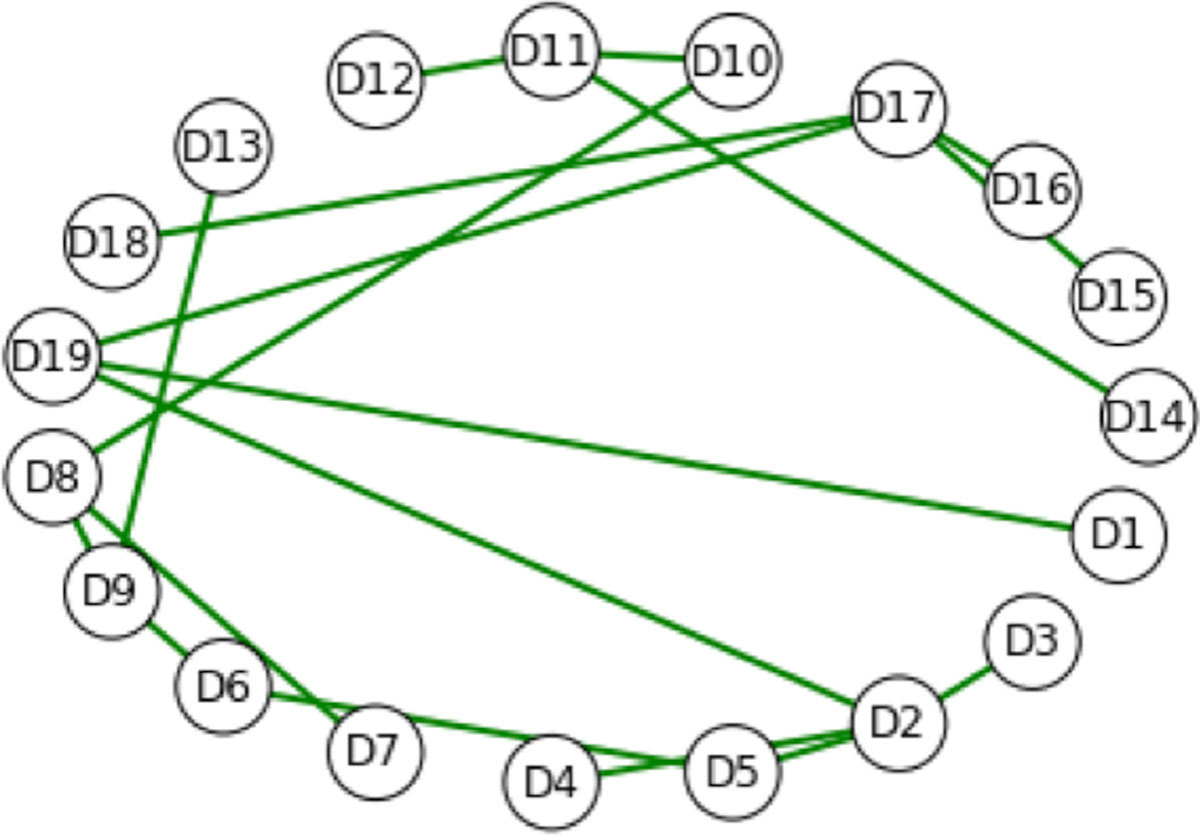
**The minimum spanning tree with mutations in D3 only**. The spanning tree is the same as in Figure 4 (no mutations), demonstrating that mutations in D3 do not change the minimum spanning tree. Additional filtering can be applied to other features to identify the differential localization of separate biophysical effects. Comparing these results to Figure 4 for the wild-type suggests that D3 is not involved in the changes to the hydropathy spanning features.

**Figure 8:**
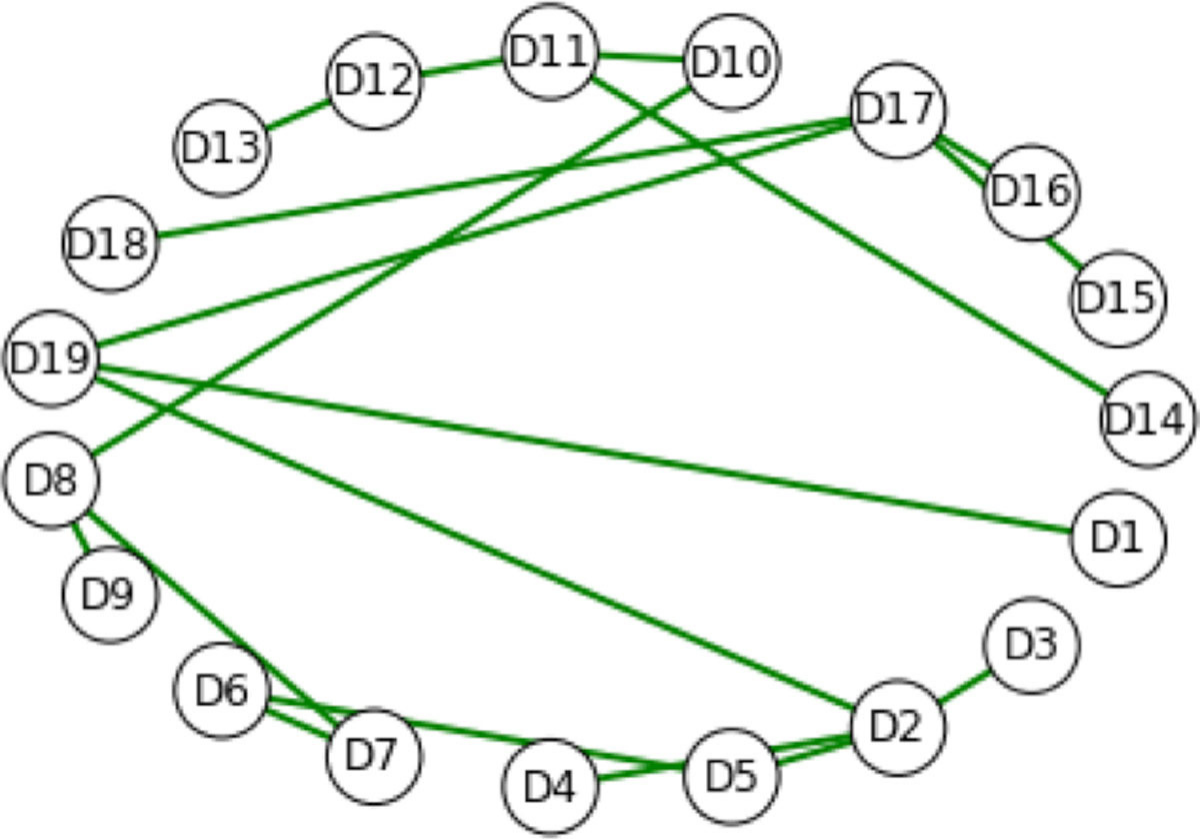
**The minimum spanning tree with mutations only in D9**. Even though only those mutations from dTIM corresponding to D9 are included, the mutations in D9 significantly alter the unique minimum spanning tree. Selecting a different domain in which to examine mutational effects shows that mutations in D9 affect the hydropathy spanning tree much more than mutations in D3..

Using different subsets of the mutations in the sequence for dTIM we obtained different spanning trees, although some vertices in the top level graph were highly conserved across the collection of mutations. Figure 4 shows the minimum spanning tree of the top level graph of scTIM, while Figure 5 shows the minimum spanning tree for the all-mutations residue sequence dTIM. For Figures 4 and 5, we used a descriptor based on hydropathy [20]. In both cases, the minimum spanning tree is unique. Figure 6 shows the mean (point) and standard deviation (error bar) across a large number of mutation resamplings, where a mutation resampling was a random selection of mutations from the defective applied to the wild type. Sample size and number of samples were chosen so that each mutation was expected to occur 2.5 times. Figure 6 implies that the degrees of vertices D3, D8, D9, D11, and D18 are unaffected by mutations, whereas structure D4, D12, D17, and D19 are highly sensitive to mutations in the residue sequence. Subsets of the mutations allow individual structure to be studied independently. For example, Figure 7 shows that if mutations are allowed only in D3, then the minimum spanning tree is essentially that of the wild type graph. In contrast, Figure 8 shows that if mutations are only allowed in D9, then the minimum spanning tree is altered.

## Discussion

As the designers of this challenge intended, a problem of great interest in the field of molecular biology and biomedical science is how a single point mutation in some instances can have virtually no effect on the structure and function of a protein while in other cases the results can be disastrous. For example, a mutation in the gene for the cystic fibrosis conductance transmembrane regulator causes the protein to misfold and be tagged for degradation [21]. Consequently, people with this mutation do not have this needed membrane protein in their epithelial cells and the result is the disease Cystic Fibrosis. We note that for most people, the mutation is a single point mutation, the deletion of phenylalanine (F) at position 508. The protein has a total of 1482 residues and thus the absence of only one residue out of the nearly 1500 residues has severe consequences. Molecular dynamics has shown the deletion of F at 508 causes very little change at the local level [22], so there must be some means by which this single deletion, i.e., a minor change at the local level percolates the entire structure.

This is the idea behind the vertex-weighted hierarchical graph model. A change at the amino acid level can be quantified on the bottom level and relayed to the mid-level by a change in vertex weights in the corresponding midlevel graph. This change in turn results in a change in the weights of the Top Level graph. The discussion about CFTR is an illustrative example of the concept and not meant to be restrictive. One could replace "deletion" with "insertion" and the discussion would remain the same in that the corresponding midlevel graph with the insertion would change and consequently the vertex representing that midlevel graph would receive a new set of descriptors, i.e, new weights. In addition, biochemical properties associated with the residues such as ss-stability and Vander Waals are included as vertex weights for the amino acids. For access to the IPython notebook and other materials, the reader may contact the authors of the paper.

## Conclusions

Not all graphical invariants are informative for every graph. For example, the connectivity number provides no discerning information on a set of trees since all trees have connectivity number 2. In the same way, it should be noted that not all descriptors can be used to infer the impact of a mutation to the residue sequence. Proteins vary widely in size and structure. Thus, in practice, results and meaningful visualizations require a careful selection and testing of candidate descriptors and vertex weighting methods. The general model however can always be applied. For each application, the size of the structure, the types of the descriptors, and even the number of levels of the hierarchical graph must be determined by the modeller. In addition, the method only works as good as the selected protein prediction software when that software is used to determine the corresponding mutant graph. Our focus here is on visualizing what the software predicts so that, at a glance, one can observe the global structural consequences of a mutation when viewed through the lens of graph theory.

We now discuss some of the specifics in the methods section.

## Methods

We implemented the hierarchical modelling process as an IPython notebook [23] running on the Python distribution *Anaconda 1.8*. This implementation begins by reading in protein three dimensional conformation data in the pdb file format via the module *biopython *[24]. A single chain in the pdb model must be selected, and then either all or sections of the chain can be used to produce the hierarchical structure. In this way a connected graph is constructed for each chain. These chain graphs can then be connected by edges based on proximity if a protein has more than one chain. Given that the contest designers only provided mutations for one of the chains of TIM, our work was restricted to that chain.

An atom-based contact map is used to generate the lowest level graph. Measures for distances between residues include Cα to Cα, between centroids, and between corresponding centers of mass. Edges are weighted with the number of contacts between two residues, and the distance measures can be all-atom or restricted to side chains. In addition to the pdb file, the notebook uses a file "AADescriptorsRaw.csv" which contains a number of amino acid descriptors and graph-theoretic measures. As described earlier, we modify a number of the standard graph-theoretic measures to incorporate the vertex weights. In particular we find weighted upper domination, weighted lower domination, weighted diameter, circumference, average weighted degree, weighted periphery which we define by generalizing standard graphical invariants. Additionally, we use Plr, Chrg, Hydpthy, stablty, ss-stability, vanderWaal, chargetransf, chargedonar, averhydrophocitiy, coilConformation, IsoElectric, Balaban index, RofGyr, ShapeIndex, EIIP to be the most informative from a long list of highly used amino acid indicies. Many of these can be found in the Amino Acid Index Database [25].

Thus, the lowest level - the all atom level - is used both to define the structure and the vertex properties of the mid-level graph. A list of ranges defines the vertices (substructures) of the mid-level, contact-map generated graphs. Each of these substructures are in turn the vertices of the top level graph. Once again, edges are defined by the contacts between the structures, with at least two contacts between substructures necessary for an edge in the top level graph. Also, the top level edges are once again weighted by the number of contacts between the substructures (which are the vertices of the top level graph.)

Graph based descriptors are defined for the substructure graphs Di and for the top level graph. For example, the maximum generalized degree of a substructure graph is the largest vertex weighted degree corresponding to a given amino acid descriptor. The vertex weighted degree over a given descriptor is the sum of the descriptor values over the neighborhood of that vertex. A substructure-wide descriptor thus provides vertex weights for the vertices in a top level graph, and these vertex weights can be used to infer properties of the top level graph. The result can be exported to graphml as a vertex weighted graph, after which a visualization tool such as *Cytoscape *can be used to visualize the impact of sequence level changes on the top level graph of a protein.

There are numerous way to quantify structural aspects of a graph and these quantities are typically called graphical invariants in graph theory. For example, if the edges of the graph are weighted, then the minimum weight of a spanning tree is a quantity that is well known and highly studied. Thus, we use the vertex weights to determine a corresponding scheme for edge weights and then utilize the fact that there exist algorithms to find the minimum spanning trees of (edge) weighted graphs. The minimum weight among all spanning trees is just one of many ways to quantify a graph and these are the quantities that can then be drawn upon as vertex weights for the next level up the hierarchy, or used as part of the final quantification of the graph if one is calculating the minimum spanning tree of the top level graph. We have explored a number of graphical invariants from graph theory and molecular descriptors from computational chemistry. This work illustrates the concept and utility of the vertex-weighted hierarchical graph as an effective modelling and visualization tool for the investigation of the consequences of a mutation.

## Competing interests

The authors declare that they have no competing interests.

## Authors' contributions

Both authors contributed equally to the project.

## Declarations

Publication of this work was supported by an East Tennessee State University Research Development Grant (#E82258).

This article has been published as part of *BMC Proceedings *Volume 8 Supplement 2, 2014: Proceedings of the 3rd Annual Symposium on Biological Data Visualization: Data Analysis and Redesign Contests. The full contents of the supplement are available online at http://www.biomedcentral.com/bmcproc/supplements/8/S2
